# Correction: Non-thermal plasma assisted surface nano-textured carboxymethyl guar gum/chitosan hydrogels for biomedical applications

**DOI:** 10.1039/c9ra90082a

**Published:** 2019-11-07

**Authors:** Ganeswar Dalei, Subhraseema Das, Smruti Prava Das

**Affiliations:** Department of Chemistry, Ravenshaw University Cuttack Odisha 753003 India subhraseema@gmail.com dassmrutiprava@yahoo.in

## Abstract

Correction for ‘Non-thermal plasma assisted surface nano-textured carboxymethyl guar gum/chitosan hydrogels for biomedical applications’ by Ganeswar Dalei *et al.*, *RSC Adv.*, 2019, **9**, 1705–1716.

The authors regret that incorrect images were mistakenly included in Fig. 7 of the original article, and in the graphical abstract. The correct versions of Fig. 7 and the graphical abstract are presented below. These changes do not affect the overall conclusions of the paper.

**Fig. 1:**
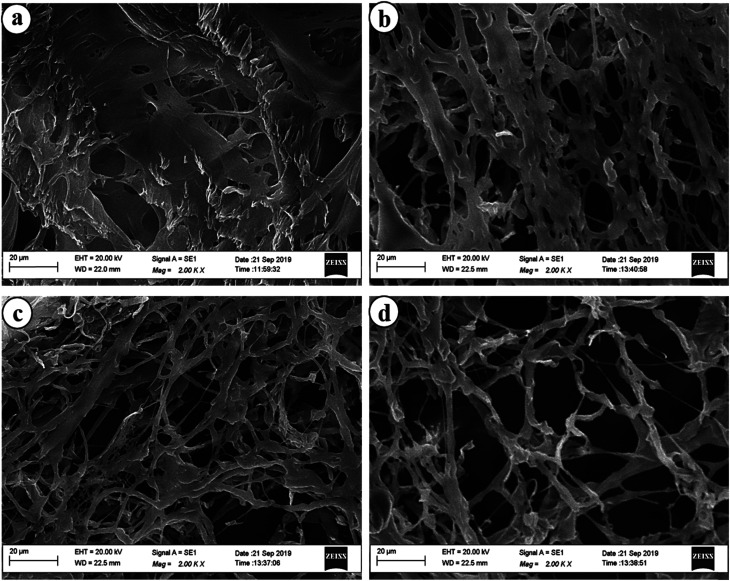
SEM images of (a) HG@UT, (b) HG@Ar, (c) HG@O_2_ and (d) HG@ Ar + O_2_.


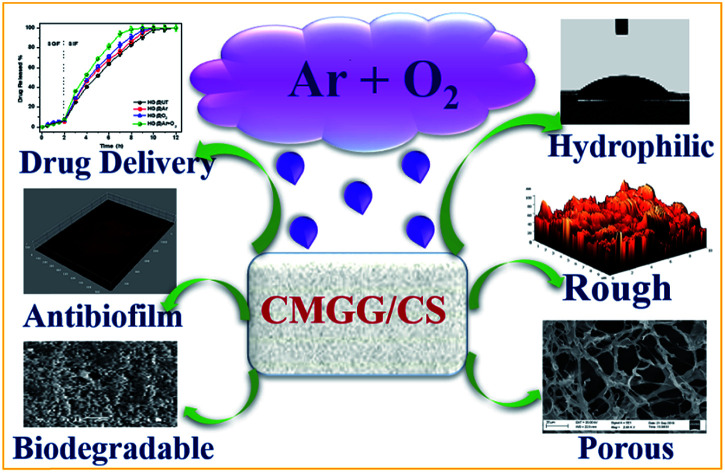
The corrected graphical abstract is presented below.

The Royal Society of Chemistry apologises for these errors and any consequent inconvenience to authors and readers.

## Supplementary Material

